# The role of reading and writing history, education, and socioeconomic status on word and pseudoword naming in adult readers of Brazilian Portuguese

**DOI:** 10.1186/s41155-026-00402-4

**Published:** 2026-07-04

**Authors:** Letícia Priscila Pacheco, Lilian Cristine Hübner, Maximiliano Agustin Wilson

**Affiliations:** 1 Present Address: Instituto Federal Sul-rio-grandense, Venâncio Aires, Brazil; 2 Pontifical Catholic University of Rio Grande do Sul, Porto Alegre, Brazil; 3 National Council for Scientific and Technological Development, Cnpq, Brasilia, DF Brazil; 4 Centre interdisciplinaire de recherche en réadaptation et intégration sociale, CIRRIS, Quebec, Canada; 5 École des Sciences de la Réadaptation, Faculté de Médecine, Université Laval, Quebec, Canada

**Keywords:** Reading history, Writing history, Lifespan, Word naming, Socioeconomic status, Cognitive reserve

## Abstract

**Background:**

Multiple factors contribute to cognitive maintenance and the development of cognitive reserve in aging, including life experiences and behaviors. Among these, education plays a key role, particularly through language-related activities such as reading and writing.

**Objective:**

This study investigates the impact of Reading and Writing Habits (RWH), alongside educational level and socioeconomic status (SES), on word and pseudoword naming across the lifespan in Brazilian Portuguese.

**Methods:**

Using a stepwise linear regression model, we analyzed the predictive value of age, SES, education, and RWH on naming performance in young and older adults, utilizing response time and accuracy as dependent variables.

**Results:**

Results indicate that age, SES, education, and RWH significantly influence naming latencies and accuracy. While education showed the strongest effect on response time, RWH also contributed meaningfully, particularly in populations with limited formal education and low SES.

**Conclusion:**

These findings suggest that RWH may serve as an important compensatory factor in cognitive processing. Overall, the study highlights the combined role of formal education and lifelong reading and writing habits in supporting language-related cognitive performance and maintaining cognitive reserve, especially in socioeconomically disadvantaged populations.

## Introduction

Environmental and behavioral factors have already been identified as contributing factors in maintaining cognition in aging, as well as predictors of cognitive decline or dementia when associated with genetic predisposition (Kim et al., 2024; Larnyo et al., 2022; Malcorra et al., 2022; Riffo et al., 2025). Developed by Stern (2009), the concept of “Cognitive Reserve” is understood as an active model, which is influenced by various aspects of daily life, such as reading habits, writing, regular physical activity, stress levels, and types of work activities performed, among other factors. Both the quantity and quality of stimuli accessed throughout life can promote a kind of reinforcement in cognition, strengthening neural connections and promoting better conditions for cognitive maintenance (Stern, 2009; Stern et al., 2019; Wulf et al., 2019). Recent research suggests that various factors can strengthen cognitive reserves in aging. This finding is associated with regular reading and writing habits throughout life, which are often stimulated by cultural, family, or professional factors. According to Pillai et al. (2012), reading and educational experiences have a significant effect on brain architecture, as people with low levels of education had smaller volumes and reduced density in the brain regions observed by the research. Life experiences, years of formal education, and reading and writing practices may be directly related to this finding, since avid readers tend to maintain or increase their lexical repertoire based on their experiences with language. One of the identified influential factors for building cognitive reserve is the number of years of formal education as a means of cognitive stimulation (Krivanek et al., 2021).

One of the significant aspects of investigating the relationship between adults and elders and education lies in the fact that education continues to exert its impact beyond adulthood and into old age (Lövdén et al., 2020). This perspective is shared by Walhovd et al. (2023), emphasizing that a person’s past is as crucial as their present state and habits when exploring their health and making predictions about future conditions. Usually, educational attainment is evaluated by observing the years of formal education a person has received in their lifetime. However, it is important to consider other factors such as the subject’s reading and writing history, as well as any previous difficulties they have faced in this regard. In a study of older adults diagnosed with subjective cognitive decline, Belleville et al. (2023) identified a strategic shift in individuals with lower education and increased expertise in those with higher education. More educated adults developed better cognitive functions, while less educated ones developed strategies to maintain their cognitive functions.

In addition to education, reading and writing habits (RWH) may be considered the most common activities related to cognitive stimulation. Reading should be understood as a dynamic process, where occupation-specific reading becomes increasingly vital during adulthood (Locher & Pfost, 2019). Recently, researchers have examined RWH as a possible contributor to cognitive reserve across the lifespan. Takeuchi et al. (2016) found in Japanese children a positive association between the strength of reading habits and verbal comprehension scores. The study suggests that good reading habits may be directly or indirectly linked to the development of working memory, which is critical to reading and language processes. In older adults, a positive history of RWH, when combined with other aspects of well-stimulated cognition, may establish favorable conditions for cognitive reserve (Karsazi et al., 2024; Kim et al., 2024; Riffo et al., 2025). This, in turn, may contribute to better maintenance of daily activities and communication during aging (Malcorra et al., 2022). Similarly, in their study with English-language university students in Ecuador, Mora et al. (2021) found that RWH may have different impacts on reading speed and comprehension. The results showed that while English language reading speed was below average, students demonstrated to have a high level of reading comprehension, which may be consistent with the additional attention dedicated to the task when satisfactory comprehension is demanded. In line with data indicating a relative slowness in aging (Burke & Shafto, 2008; De Beni et al., 2007; Deery et al., 2023; Grigorenko & Naples, 2011), these studies demonstrate better comprehension without maintaining the usual processing speed. Furthermore, previous studies conducted with English and French word and pseudoword naming (Chapleau et al., 2017; Provost et al., 2016; Wilson et al., 2012) observed that typical older adults name regular and irregular words with longer latencies compared to young adults, while maintaining accuracy. This suggests that the reading experience may influence the quality of word naming, while aging-related slowness may influence latencies.

A reliable assessment of RWH through a comprehensive tool is necessary to organize a study focusing on the effect of those habits on specific cognitive abilities. To assess RWH, one of the most widely used tools is the Adult Reading History Questionnaire (ARHQ; Lefly & Pennington, 2000). The ARHQ is a diagnostic tool for screening reading disabilities. The questionnaire was found to be reliable in diagnosing reading disability and showed substantial incremental validity in a hierarchical analysis that initially included IQ and SES (Lefly & Pennington, 2000), which demonstrates its usefulness for investigations involving these measures. The questionnaire’s significance lies in tracing a person’s reading habits and attitudes towards reading throughout their life, as a person’s history of reading and education during childhood can affect their quality of learning during their school years (Gilger et al., 1991; Krasowicz-Kupis et al., 2014).

Lefly and Pennington’s, 2000 questionnaire has been adapted to several languages to aid in identifying dyslexia in adults. One such study was performed by Bjornsdottir et al. (2014), which analyzed the Icelandic version’s reliability and psychometric properties. Welcome and Meza (2019) examined the structure of the questionnaire using both exploratory and confirmatory factor analyses. Their study identified subscales that captured specific latent factors and demonstrated associations between the resulting scores and multiple reading-related abilities. These findings suggest that reading history is a multidimensional construct that warrants comprehensive investigation in future research. This framework provides a methodological basis for addressing the different ways in which the ARHQ questionnaire captures participants’ experiences and how the resulting scores can be integrated into analyses. Depending on the research objectives, scores may be considered in their entirety or selectively, focusing on particular items or subscales.

In summary, existing literature highlights that robust Reading and Writing Habits (RWH) are consistently associated with enhanced working memory, reading comprehension, and the preservation of global cognitive architecture. However, a critical gap remains: most studies have focused on highly educated populations in developed nations. This leaves a weak spot in our understanding of how RWH operates as a compensatory mechanism in developing countries with high socioeconomic and educational heterogeneity. Furthermore, measuring reading history is a complex construct. Tools like the Adult Reading History Questionnaire (ARHQ; Lefly & Pennington, 2000) address this by capturing both current reading habits and early childhood reading history. Interestingly, the ARHQ serves a dual purpose: tracing lifelong habits and identifying reading disabilities like dyslexia. The interaction between these two factors is deeply intertwined; early struggles with literacy acquisition (such as dyslexia) profoundly shape an individual’s attitudes toward reading, often leading to avoidance behaviors that restrict their lifelong RWH and, consequently, their cognitive reserve.

In Brazilian Portuguese, RWH can be measured by means of the Reading and Writing Habits questionnaire from the *Bateria de Avaliação da Linguagem no Envelhecimento (in English*,* Language Assessment Battery in Aging)* (BALE; Hübner et al., 2019). The BALE has been used to examine the impact of RWH on typical and atypical aging. Malcorra et al. (2022) examined the role of age, education, RWH in normal conversation in spoken language (speech connectedness). The study revealed that the combination of age, education, and RWH accounted for improved speech connectedness. Schneider et al. (2022) investigated the relationships among macrolinguistic, neuropsychological, and sociodemographic factors in stroke patients with low levels of education and socioeconomic status, both with and without left- or right-hemisphere lesions. Their results indicated that stronger macrolinguistic abilities—defined as the capacity to organize ideas at the global level of discourse—were positively associated with regular reading and writing habits.

Given that the BALE is a brief questionnaire, additional details are required for a more comprehensive assessment of reading and writing history (RWH), including both past and current habits as well as difficulties encountered during literacy acquisition. We therefore propose that a questionnaire written in Brazilian Portuguese (BP), inspired by both the ARHQ and BALE, which will be described in the section Questionnaire of Reading and Writing Habits for Adults (QHLEA-JI), could provide a more reliable tool for collecting data within the Brazilian population. Moreover, as the BALE yields scores on a scale of up to 32 points, expanding the score range should be considered, since statistical analyses rely on more complete and fine-grained data to more accurately capture population-level patterns (Rasinger, 2010).

Building on this rationale, the present study investigates the reading histories of healthy young and older Brazilian adults to examine the role of RWH and educational background in word and pseudoword naming. This work is contextualized within prior research on word and pseudoword naming in English and French (Chapleau et al., 2017; Provost et al., 2016; Wilson et al., 2012). To extend these findings, we conducted regression analyses with a diverse sample of Brazilian Portuguese speakers, aged 18 to 85 years, representing a range of socioeconomic statuses (SES) and RWH profiles.

## Method

This study was approved by the PUCRS Ethics Committee under CAAE 53696221.4.1001.5336. The declaration of consent for voluntary participation of the research subjects was collected with the “Term of Free and Informed Consent – Behavioral”.

### Participants

Seventy healthy Brazilian Portuguese-speaking adults, aged between 18 and 85 years (M = 45.5, SD = 15.53), with varying levels of education (M = 12.79, SD = 15.27) and socioeconomic status (SES) (M = 3.15, SD = 1.24), participated in the study. To accurately capture the developmental trajectory of naming performance across the adult lifespan, participants were assessed as a single, continuous cohort rather than being divided into discrete, categorical age groups (e.g., “young” versus “old”). This single-group design was deliberately chosen to align with our regression-based analytical approach, maximizing statistical power by treating age, education, and socioeconomic status (SES) as continuous predictor variables.

Socioeconomic status (SES) (M = 3.15, SD = 1.24) was carefully documented. Because SES distribution varies greatly across Brazil, it is important to note that data collection took place in the state of Rio Grande do Sul. This region reflects a specific socioeconomic reality within the broader national context, which was captured using the ABEP (2021) classification. The participants had normal or corrected-to-normal vision, without a history of depression or any neurological pathologies. They were also informed of the risks and benefits of the protocol used and that they could quit the tasks at any time.

### Instruments

#### Questionnaire of reading and writing habits for adults – young and older adults – in BP

The self-reported history of reading difficulties and reading habits has been used to provide data on the quantity and quality of reading throughout life. Previous research has shown the reliability of adult self-reported reading skills when studying a substantial number of participants (Gilger, 1992). To measure RWH in this study, we developed a reading history questionnaire for Brazilian Portuguese (BP) - QHLEA-JI (Questionário de Hábitos de leitura e escrita para adultos - jovens e idosos – in BP). The QHLEA-JI is based on excerpts of the original ARHQ by Lefly and Pennington (2000) and expands the questionnaire from BALE (Hübner et al., 2019), in order to have more comprehensive data on the participants’ reading and writing history, adapted to Brazil. It is composed of 31 items in a Likert scale format, each question allowing for possible responses rated from 0 to 4. In general, “0” responses account for frequent RWH, while “4” accounts for low frequency or absent habits. The questions tap into attitudes towards reading and writing past and present characteristics. Responses on the lower end of the scale indicate a stronger RWH throughout one’s life.

#### Socioeconomic status questionnaire (ABEP, 2021)

The questionnaire applied in this study assessed socioeconomic status (SES) using multiple indicators, including household assets (e.g., appliances, cars), number of bathrooms, presence of domestic workers, type of street pavement, access to basic sanitation, source of drinking water, and the educational level of the primary income provider. Each item contributed to a cumulative score, which was subsequently used to classify households into SES categories, ranging from the lowest (DE) to the highest (A). According to Associação Brasileira de Empresas de Pesquisa (2021), in 2024 only 3.1% of Brazilian families were classified as category A, while 28.4% fell into category DE.

#### Word and pseudoword naming

The word and pseudoword naming tasks built for this study were developed according to the criteria adopted by Wilson et al. (2012), in a study in French language reading by adults. We adapted their criteria to the characteristics of BP, using corpora available in the Brazilian Portuguese Lexicon (Estivalet, 2019) and in the psycholinguistic criteria database (dos Santos et al., 2017). The choice of word and pseudoword naming tasks—measuring both response times (RTs) and accuracy—was strategic. Beyond their widespread use in prior literature, these measures are highly sensitive behavioral indicators of lexical access and phonological decoding efficiency. Response times provide a direct window into processing speed, which typically slows with age, while accuracy reflects the integrity of the lexical repertoire, which is directly nurtured by lifelong RWH.

For the word naming task 144 words were listed, being 72 consistent for the conversion from graphemes to phonemes in PB, and 72 inconsistent, according to the description of PB structure provided by Parente et al. (1997). In each of these groups (consistent and inconsistent) there were 36 high frequency words (> 5 per million of occurrences) and 36 low frequency ones (< 5 per million of occurrences), according to LexPorBR (Estivalet, 2019). All subgroups of words were statistically balanced for initial phoneme, number of letters, phonemes and syllables, bigrams frequency, neighborhood size (Estivalet, 2019), concreteness, imageability (dos Santos et al., 2017).

The pseudoword naming task is composed of 72 pseudowords which were created from part of the words used in the naming task, changing at least one of the letters and phonemes of each word. The pseudowords were also statistically paired by initial phoneme, number of letters, phoneme and syllables, and bigrams frequency (Table 2).

#### Education

Education was measured based on participants’ self-reported years of formal schooling, excluding any repeated grades or incomplete academic years.

### Procedure

All tasks were completed in a single session lasting approximately 40 min. At the outset, participants provided informed consent. Task order was counterbalanced: half of the participants completed the Word Naming task followed by the Pseudoword Naming task, while the other half completed them in the reverse order.

Stimuli were presented on a PC using DMDX software (Forster & Forster, 2003). Participants read aloud 72 pseudowords, divided into two blocks of 36 stimuli each, followed by consistent and inconsistent words combined into four blocks of 36 items each. Both block order and stimulus presentation within blocks were randomized. Each trial began with a 400 ms blank screen, followed by a 400 ms central fixation point. The target stimulus (word or pseudoword) was then displayed in lowercase Arial font (15 pt) for up to 1,500 ms.

Each experimental session began with a practice set of 10 items (pseudowords or words, depending on the assigned order). Participants were instructed to read each stimulus aloud as quickly and accurately as possible. Accuracy and response times (RTs) were extracted from recorded responses using the CheckVocal program (Protopapas, 2007), which captures vocal responses audiovisually, generating waveforms, spectrograms, and audio recordings.

The subsequent task was the QHLEA-JI, administered via a PC interface. Participants read the questions presented on a Google Form and registered their answers in a 0–5 scale. Following this, participants completed the Socioeconomic Status Questionnaire (ABEP, 2021), which was appended at the end of the digital protocol. Questionnaires were either self-administered by the participants or, in cases where individuals were unfamiliar with digital technologies, completed with the researcher’s assistance. All items were presented in a fixed sequence, and no time constraints were imposed for responses.

### Data analysis

We first inspected the data for collinearity. Residues were normally distributed with skewness values between ± 2 in absolute value (Schmider et al., 2010) (see Table 1). We also inspected the data for multicollinearity. To this end, we ran a regression model entering RTs as a dependent variable and all the others as independent variables. We calculated the tolerance and variance inflation factor (VIF). The results showed VIF < 4 and tolerance > 0.2 for all variables (see Table 1). This suggests the absence of multicollinearity. All statistical analyses were performed using Jamovi software (2023).

**Table 1:** Summary statistics for the predictor variables and the word naming reaction times and scores for word and pseudowords

Type of Stimuli	Predictor Variable	Mean	Standard Deviation	Range	Skewness	Tolerance	VIF
Word	Age	45.543	15.292	18–85	0.5515	0.923	1.08
	SES	3.157	1.249	1–6	0.3605	0.679	1.47
	RWH	43.800	18.536	11–89	0.3079	0.842	1.19
	Education	12.771	6.479	3–27	0.0698	0.602	1.66
	RTs	843.117	188.268	451–1475	0.83502	n/a	n/a
	Score	0.899	0.302	0–1	0.31175	n/a	n/a
Pseudoword	Age	45.543	15.293	18–85	0.5516	0.930	1.08
	SES	3.157	1.250	1–6	0.3606	0.696	1.44
	RWH	43.800	18.537	11–89	0.3080	0.855	1.17
	Education	12.771	6.480	3–27	0.0698	0.625	1.60
	RTs	929.550	200.843	463–1475	n/a	n/a	n/a
	Score	0.887	0.317	0–1	0.5049	n/a	n/a

Age, age of participants; SES, Socioeconomic Status (ABEP, 2021); RWH, Reading and Writing habits; Education, years of formal schooling; RTs, word and pseudoword naming reaction times; Score, word and pseudoword naming scores

We conducted a linear regression for RTs and a binary logistic regression for score as dependent variables. Rather than dichotomizing the sample into separate age groups—which can lead to a loss of variance and statistical information—age (in years) was entered into the models as a continuous variable alongside SES and education. We introduced two steps in the model. In the first step of both models, age (in years), socioeconomic status (SES), and education (in years) were entered as predictors. Age was included due to our focus on developmental trajectories of word naming across the lifespan, as prior research has demonstrated age-related changes in word naming performance across different languages (Newman & German, 2005; Spieler & Balota, 2000). SES was incorporated given its particular relevance in the context of developing countries, such as Brazil—the focus of the present study—where socioeconomic disparities may significantly influence cognitive and linguistic outcomes (Bonifacci et al. 2024; Salas and Pascual 2023; Tan 2024a, b). Education was included due to its well-established role in reading proficiency and broader language-related abilities across the lifespan (Lövdén et al., 2020; Steffener, 2020a). In the second and final step, we entered RWH, as our primary aim was to assess the extent to which RWH predicts word and pseudoword naming latencies. Logistic regression models were fitted using the maximum likelihood estimation method (McFadden’s pseudo R²).

## Results

For word naming RTs, age, education, and RWH were all significant predictors of word naming, all *p*s < 0.001. SES was also a significant predictor, however with *p* = .003. Step 2 accounted for a significant increase in the variance (*R*² = 0.15, Δ*R*² = 0.003, *F*(1, 9311) = 401.00, *p* < .001), also observing RWH as a predictor variable for RTs in word naming (*p* < .001). Older participants presented longer RTs. Participants in more vulnerable SES brackets were slower in word naming. More educated participants had shorter RTs, and participants with stronger RWH were faster in word naming. Table 2 shows the results of the regression models.

**Table 2:** Standardized coefficients from the regression analyses for latencies and accuracy in word and pseudoword naming

	Type of stimuli			
	Word		Pseudoword	
	RT	Score	RT	Score
*Step 1*				
Age	1.02***	-0.04***	0.42*	-0.04***
SES	5.01***	0.16***	-0.43	0.00
Education	-9.33***	0.18***	-11.14***	0.20***
R² / R² mcf	0.164	0.191	0.124	0.23
*Step 2*				
RWH	1.91***	-0.03***	18.08***	-0.02***
R² / R² mcf	0.20	0.22	0.14	0.24
ΔR² / χ²	0.035***	205***	0.021***	61.4***

Columns refer to the *Β*, and their titles show the dependent variables of the model (RTs / Score). ΔR² / χ² are the incremental increase in the model R2 that results from the addition of RWH as a predictor in Step 2 of the model

*p < .05, **p < .01, and ***p < .001

For word naming accuracy, age, SES, and education (*p* < .001) were found to be significant predictors. Younger and more educated participants presented higher scores as compared to older and less educated ones. The same pattern was observed for SES and education: participants with higher SES and more years of formal education were more accurate in the word naming task. Step 2 accounted for a significant increase in the variance (*R*² = 0.23, *χ*²(1, *N* = 70) = 205.00, *p* < .001). RWH was also a significant predictor (*p* < .001). However, participants with stronger RWH presented lower scores in the word naming task as compared to those with lower RWH .

For pseudoword naming RTs, age was a significant predictor (*p* = .03), followed by education (*p* < .001). Younger participants presented shorter RTs as compared to older ones, and more educated participants presented shorter RTs as well. SES was not a significant predictor (*p* = .87). Step 2 accounted for a significant increase in the variance (*R*² = 0.15, Δ*R*² = 0.021, *F*(1, 4462) = 110.00, *p* < .001). RWH significantly explained variance in RTs (*p* < .001), with participants reporting more frequent RWH showing shorter RTs than those with less frequent RWH.

**Fig. 1:**
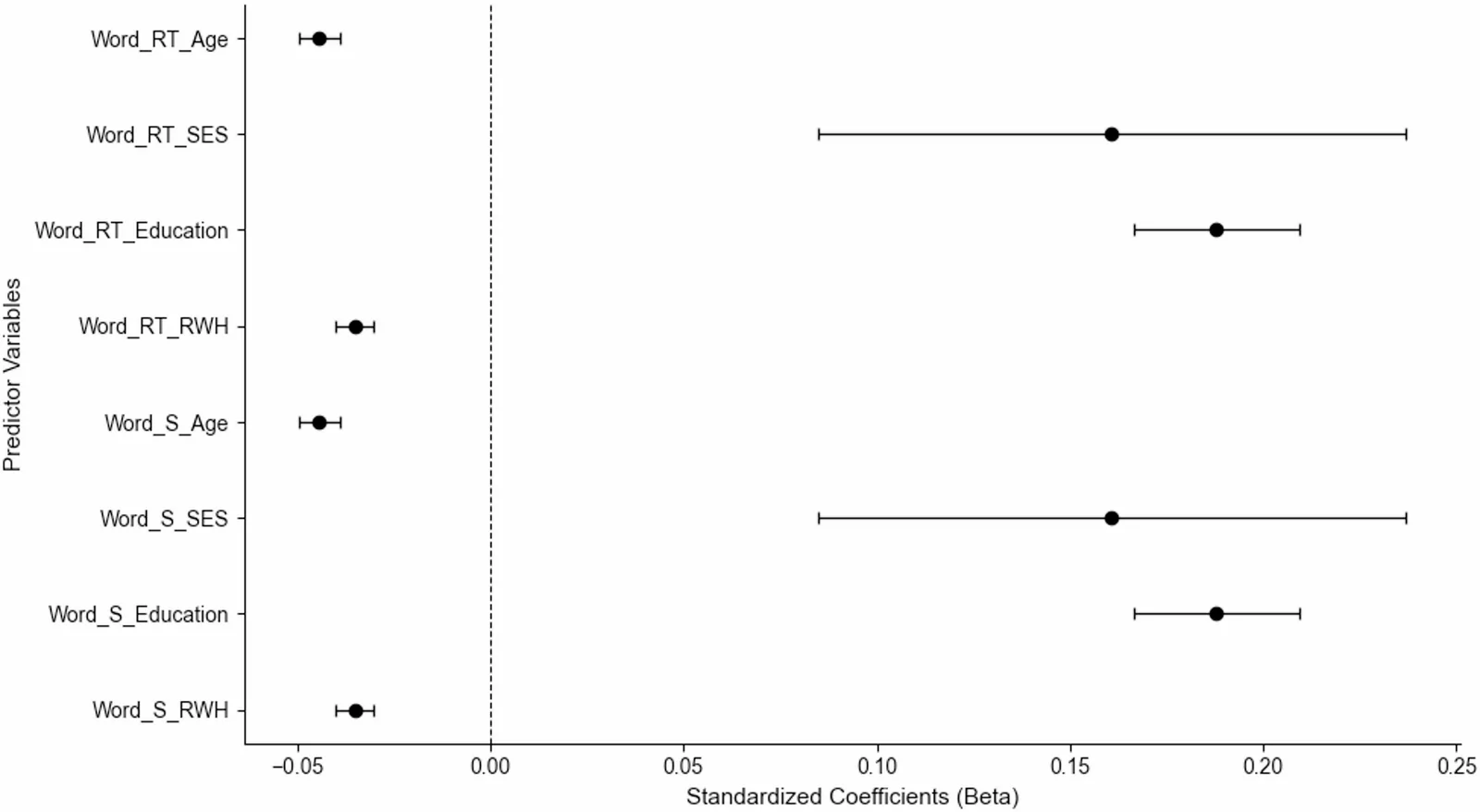
Standardized beta coefficients and 95% confidence intervals for word naming predictors. Note. Black circles represent standardized beta estimates. Horizontal error bars represent 95% confidence intervals. Predictors with confidence intervals that do not cross the vertical dashed line (zero) indicate statistical significance at the .05 level. RT = Response Time; S = Score; SES = Socioeconomic Status; RWH = Reading/Writing Habits

**Fig. 2:**
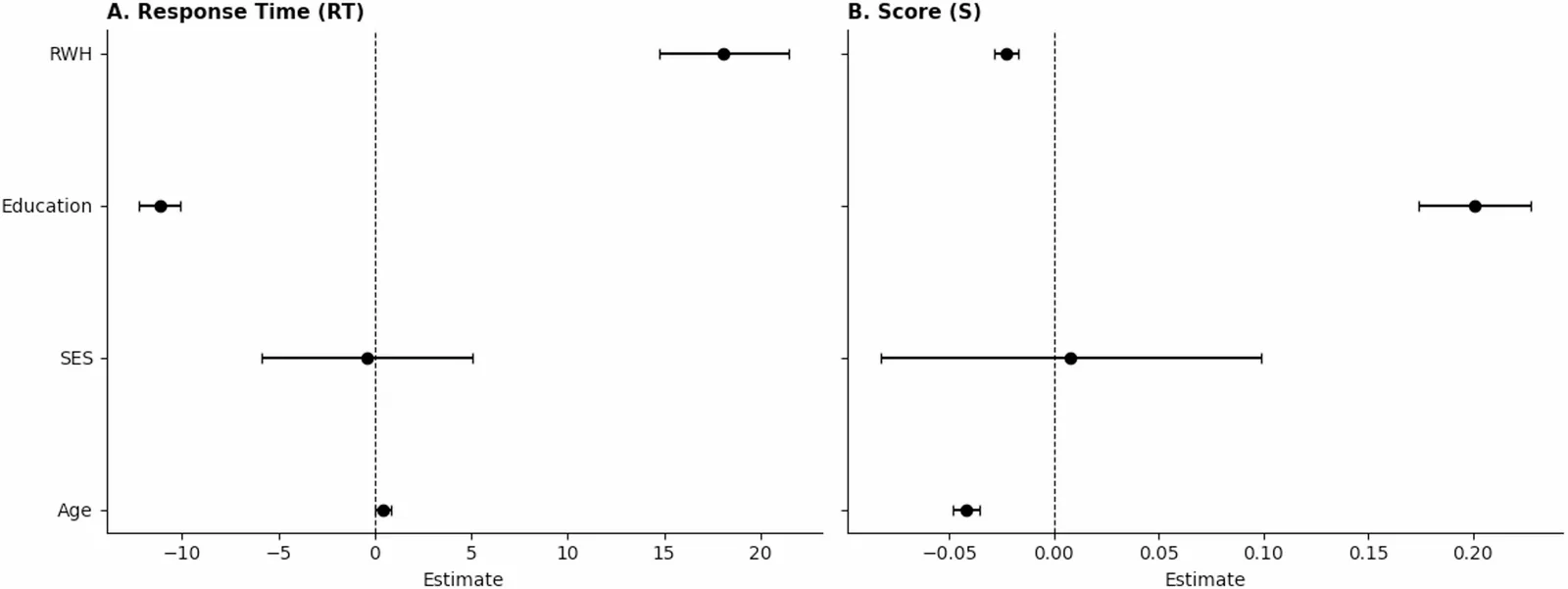
Standardized beta coefficients and 95% confidence intervals for pseudoword (Pw) processing predictors. Note. Panel A shows the results for Response Time (RT), and Panel B shows the results for Score (S). Black circles represent standardized beta estimates, while horizontal error bars indicate 95% confidence intervals. Predictors whose confidence intervals do not cross the vertical dashed line (zero) are considered statistically significant at the .05 level. SES = Socioeconomic Status; RWH = Reading/Writing Habits

For pseudoword naming accuracy, both age and education emerged as significant predictors (*p* < .001). Younger participants and those with higher levels of education performed more accurately than older and less educated participants. SES was not a significant predictor (*p* = .862). Step 2 accounted for a significant increase in the variance (*R*² = 0.25, *χ*²(1, *N* = 70) = 61.40, *p* < .001). Even though RWH was a significant predictor (*p* < .001), unlike what was expected, participants with more frequent RWH had lower scores for pseudoword naming (Figs. 1 and 2).

## Discussion

This study investigated the role of reading and writing habits (RWH) and education in word and pseudoword naming performance among younger and older adult speakers of Brazilian Portuguese. It is important to highlight the fact that we have investigated word naming skills of participants, which may not fully grasp participants’ reading skills as in tasks which measure text comprehension performance. Focused on word naming, our findings indicate that age significantly influenced both latencies and accuracy: older participants exhibited slower responses and lower accuracy compared to younger adults. These results provide novel evidence for word naming in aging, as prior studies in French and English have reported greater accuracy among older adults than among younger ones (Chapleau et al., 2017; Joyal 2017; Provost et al., 2016).

Regarding pseudoword naming, age effects were observed in accuracy but not in response times. Younger adults achieved higher accuracy than older adults, but no age-related differences emerged in latencies. This pattern diverges from findings in French (Chapleau et al., 2017), where older adults were both faster and more accurate than younger ones. Further research is needed to clarify the sources of slower responses and increased error rates among Brazilian older adults. One possible explanation lies in the historically limited reading culture in Brazil (Retratos da Leitura no Brasil, 2024) and the comparatively recent development of national educational policies (Brasil, 1996), coupled with the poor schooling conditions faced by many members of the older generation during their formative years.

Socioeconomic status (SES) also emerged as a significant predictor of word, though not pseudoword, naming latencies: participants with higher SES demonstrated shorter response times. To our knowledge, no previous studies have directly examined SES effects on word and pseudoword naming, underscoring the importance of including this variable in studies of language and cognition—particularly in underdeveloped countries, where SES distributions tend to be more heterogeneous and skewed toward lower levels (Larnyo et al., 2022). SES is increasingly recognized as a contributor to cognitive reserve in aging (Brewster et al., 2014; Krueger et al., 2025; Wang, 2023, Wang et al., 2023). In the present study, however, SES did not predict pseudoword naming latencies or accuracy.

Education, by contrast, exerted robust effects across tasks. Higher educational attainment was associated with greater word naming accuracy and shorter pseudoword naming latencies, as well as higher pseudoword naming accuracy. These findings converge with extensive evidence identifying education as a consistent predictor of reading performance across multiple tasks, including word fluency, reading comprehension, literacy assessments, vocabulary encoding and decoding, and neuroimaging outcomes (Mellard et al., 2010, 2015; Lövdén et al., 2020; Rakesh 2024; Rakesh et al., 2024; Steffener, 2020b). Prior studies have also demonstrated the influence of years of education on naming performance (Ashaie & Obler, 2014; Le Dorze & Durocher, 1992). Beyond behavioral data, neuroimaging studies suggest that education supports the preservation of white and gray matter integrity, thereby contributing to the maintenance of general cognitive functions in aging, including language (Takeuchi et al., 2016; Sörman et al., 2018). Takeuchi et al. (2016) further observed a positive association between reading habits and verbal comprehension scores in both cross-sectional and longitudinal analyses, while Sörman et al. (2018) reported a lasting relationship between book reading and cognitive ability across the adult lifespan.

RWH also emerged as a significant predictor of both response times (RTs) and accuracy in word and pseudoword naming. Lower scores on the questionnaire indicate a more frequent and stable engagement in RWH across the lifespan, which in turn was associated with faster latencies and greater accuracy in both tasks. Consistent with this finding, prior studies have demonstrated the positive impact of RWH on a range of language abilities, including picture naming (Ashaie & Obler, 2014), second-language learning and training (Belleville et al., 2023), and oral narrative production (Malcorra et al., 2022). Importantly, RWH exerted distinct effects on pseudoword naming: participants with more frequent habits exhibited shorter RTs, yet paradoxically produced more errors. These errors were primarily lexicalizations, in which pseudowords were read as existing words. According to dual-route models of reading (Coltheart, 1978; Coltheart et al., 2001), skilled readers preferentially rely on the lexical route, accessing stored orthographic or phonological representations, rather than the non-lexical route. This reliance may explain the higher rates of lexicalization errors observed among older and more experienced readers in our sample. Supporting this interpretation, Content and Peereman (1992) and Kwok et al. (2017) reported that frequent readers are more likely to lexicalize pseudowords during naming tasks, reflecting the influence of their expanded lexical repertoire.

Further, these results align with the Word Superiority Effect, which posits that real words are processed more quickly and accurately than pseudowords. As reading experience accumulates across the lifespan, pseudoword pronunciation becomes increasingly influenced by existing words with similar orthographic patterns (Martínez-Tomás et al. 2025a, b). Caravolas (2018) similarly demonstrated that the lexicality effect strengthens in both low- and high-consistency orthographies, reflecting readers’ long-term experience and lexical consolidation.

## Conclusion

This study aimed to examine the contributions of reading and writing habits (RWH) and educational attainment to word and pseudoword naming performance in young and older adult readers of Brazilian Portuguese. Both education and RWH significantly predicted variability in naming latencies and accuracy, highlighting their potential role in cognitive reserve. The acquisition and sustained practice of reading and writing skills support language abilities throughout the lifespan. Education and RWH, alongside other factors that contribute to cognitive reserve—such as healthy lifestyle behaviors—may buffer against the negative effects of genetic risk factors and age-related cognitive decline. These findings underscore the importance of sustained investment in educational initiatives, spanning early childhood through higher education and continuing into adulthood, as a public policy priority. Such investments are likely to promote individual cognitive and linguistic development while simultaneously alleviating pressures on public health systems and social security by supporting healthier aging trajectories. Future research should further investigate the mechanisms through which education and RWH influence cognitive functioning across the lifespan, particularly regarding structural and functional neural changes associated with language processing and aging.

## Data Availability

Data available from the corresponding author on reasonable request. (https://link.springer.com/journal/10936/submission-guidelines?code=30b87695-5d55-4076-9553-5bf61ea3b714&error=cookies_not_supported&utm_source=chatgpt.com).

## References

[CR1] Ashaie, S., & Obler, L. (2014). Effect of age, education, and bilingualism on confrontation naming in older illiterate and low-educated populations. *Behavioural Neurology*, *2014*(1). 10.1155/2014/970520 PMC4006605 24825965

[CR2] Associação Brasileira de Empresas de Pesquisa (ABEP). (2021). *Critério Brasil de classificação econômica 2021*. http://www.abep.org/wp-content/uploads/2024/01/01_cceb_2021.pdf

[CR3] Belleville, S., Mellah, S., Boller, B., & Ouellet, É. (2023). Activation changes induced by cognitive training are consistent with improved cognitive reserve in older adults with subjective cognitive decline. *Neurobiology of Aging*, *121*, 107–118. 10.1016/j.neurobiolaging.2022.10.010 36401900

[CR4] Bjornsdottir, G., Halldorsson, J. G., Steinberg, S., Hansdottir, I., Kristjansson, K., Stefansson, H., & Stefansson, K. (2014). The Adult Reading History Questionnaire (ARHQ) in Icelandic: Psychometric properties and factor structure. *Journal of Learning Disabilities*, *47*(6), 532–542. 10.1177/0022219413478662 23456983

[CR5] Bonifacci, P., Ravaldini, V., Cangelosi, M., & Tobia, V. (2024). Socioeconomic status, linguistic skills and language background differentially relate to preschoolers’ emotional and behavioural profile. *Child and Adolescent Psychiatry and Mental Health*, *18*(1). 10.1186/s13034-024-00823-y PMC11490048 39425196

[CR6] Brasil. (1996). *Lei nº 9.394, de 20 de dezembro de 1996. Estabelece as diretrizes e bases da educação nacional*. Diário Oficial da União.

[CR7] Brewster, P. W., Melrose, R. J., Marquine, M. J., Johnson, J. K., Napoles, A., MacKay-Brandt, A., & Mungas, D. (2014). Life experience and demographic influences on cognitive function in older adults. *Neuropsychology*, *28*(6), 846. 10.1037/neu0000098 PMC4227962 24933483

[CR8] Burke, D., & Shafto, M. (2008). Language and aging. In *The handbook of aging and cognition* (Vol. 3, pp. 373–443). Psychology Press. *[Nota: Confirme os editores deste livro e insira-os antes do título, se aplicável, ex: In A. Editor (Ed.), The handbook.*

[CR9] Caravolas, M. (2018). Growth of word and pseudoword reading efficiency in alphabetic orthographies: Impact of consistency. *Journal of Learning Disabilities*, *51*(5), 422–433. 10.1177/0022219417718197 28720028

[CR10] Chapleau, M., Wilson, M. A., Potvin, K., Harvey-Langton, A., Montembeault, M., & Brambati, S. M. (2017). Word reading aloud skills: Their positive redefinition through ageing. *Journal of Research in Reading*, *40*(3), 297–312.

[CR11] Coltheart, M. (1978). Lexical access in simple reading tasks. In *Strategies of information processing* (pp. 151–216). *[Nota: Falta o nome do editor do livro e a editora (publisher) no final]*.

[CR12] Coltheart, M., Rastle, K., Perry, C., Langdon, R., & Ziegler, J. (2001). DRC: A dual route cascaded model of visual word recognition and reading aloud. *Psychological Review*, *108*(1), 204–256. 10.1037/0033-295x.108.1.204 11212628

[CR13] Content, A., & Peereman, R. (1992). Single and multiple process models of print to speech conversion. In J. Alegria, D. Holender, J. Junca de Morais, & M. Radeau (Eds.), *Analytic approaches to human cognition* (pp. 213–236). North-Holland.

[CR14] De Beni, R., Palladino, P., Borella, E., & Loaiza, S. (2007). Reading comprehension in aging: The role of working memory and metacomprehension. *Aging Neuropsychology and Cognition*, *14*(2), 189–212. 10.1080/13825580500229213 17364380

[CR15] Deery, H. A., Di Paolo, R., Moran, C., Egan, G. F., & Jamadar, S. D. (2023). The older adult brain is less modular, more integrated, and less efficient at rest: A systematic review of large-scale resting-state functional brain networks in aging. *Psychophysiology*, *60*. 10.1111/psyp.14159. Article e14159. PMC10909558 36106762

[CR16] Dos Santos, C. H. (2017). Critérios psicolinguísticos para a seleção de estímulos verbais no Português Brasileiro. *Psico-USF, 22*(2). *[Nota*: Na *APA 7, deve listar os nomes de até 20 autores na referência. Se possível, substitua et al. pelos nomes completos dos restantes autores. Falta também a indicação das páginas]*.

[CR17] Estivalet, G. L. (2019). *Lexicon-PT: A database of word properties for Brazilian Portuguese* [Doctoral dissertation]. *[Nota: Adicione o nome da universidade onde a tese foi defendida no final do parêntese reto e depois a base de dados ou arquivo]*.

[CR18] Forster, K. I., & Forster, J. C. (2003). DMDX: A Windows display program with millisecond accuracy. *Behavior Research Methods Instruments & Computers*, *35*(1), 116–124. 10.3758/BF03195503 12723786

[CR19] Gilger, J. W. (1992). Using self-report and parental report survey data to assess past and present academic achievement of adults and children. *Journal of Applied Developmental Psychology*, *3*, 235–256. 10.1016/0193-3973(92)90031-C

[CR20] Gilger, J. W., Pennington, B. F., & DeFries, J. C. (1991). Risk for reading disability as a function of parental history in three family studies. In *Reading disabilities: Genetic and neurological influences* (pp. 17–29). *[Nota: Falta o nome dos editores do livro e a editora]*.

[CR21] Grigorenko, E. L., & Naples, A. J. (2011). The devil is in the details: Decoding the genetics of reading. In *How children learn to read* (pp. 135–150). Psychology Press. *[Nota: Falta o nome dos editores do livro]*.

[CR22] Hübner, L. C., Loureiro, F., Smidarle, A. D., & Tessaro, B. (2019). Bateria de Avaliação da Linguagem no Envelhecimento – BALE. In N. Zimmermann, F. Deleaere, & R. P. Fonseca (Eds.), *Tarefas de avaliação neuropsicológica*. Memnon.

[CR23] Instituto Pró-Livro (2024). *Retratos da leitura no Brasil* (6th ed.). IPL. https://www.prolivro.org.br/wp-content/uploads/2024/11/Apresentac%CC%A7a%CC%83o_Retratos_da_Leitura_2024_13-11_SITE.pdf

[CR24] Joyal, M. (2017). The impact of cognitive reserve on age-related cognitive decline: A 5-year follow-up. *Aging, Neuropsychology, and Cognition*. *[Nota: Tal como acima, liste até 20 autores e adicione o volume/páginas/DOI se existirem]*.

[CR25] Karsazi, H., Rezapour, T., Ghamsari, A. S. M., Kormi-Nouri, R., & Hatami, J. (2024). Which intellectual activities are related to cognitive reserve? Introduction and testing a three-dimensional model. *Psychological Research Psychologische Forschung*, *88*(4), 1081–1091. 10.1007/s00426-024-01926-z 38315217

[CR26] Kim, Y., Stern, Y., Seo, S. W., Na, D. L., Jang, J. W., & Jang, H. (2024). Factors associated with cognitive reserve according to education level. *Alzheimer’s & Dementia*, *20*, 7686–7697. 10.1002/alz.14236 PMC11567866 39254221

[CR27] Krasowicz-Kupis, G., Bogdanowicz, K. M., & Wiejak, K. (2014). Familial risk of dyslexia in Polish first grade pupils based on the ARHQ-PL Questionnaire. *Health Psychology Report*, *2*(4), 237–246. 10.5114/hpr.2014.46235

[CR28] Krivanek, T. J., Gale, S. A., McFeeley, B. M., Nicastri, C. M., & Daffner, K. R. (2021). Promoting successful cognitive aging: A ten-year update. *Journal of Alzheimer’s Disease*, *81*(3), 871–920. 10.3233/JAD-201462 PMC8293659 33935078

[CR29] Krueger, K. R., Desai, P., & Beck, T. (2025). Lifetime socioeconomic status, cognitive decline, and brain characteristics. *JAMA Network Open*, *8*(2). 10.1001/jamanetworkopen.2024.61208. Article e2461208. PMC11846010 39982722

[CR30] Kwok, R. K., Cuetos, F., Avdyli, R., & Ellis, A. W. (2017). Reading and lexicalization in opaque and transparent orthographies: Word naming and word learning in English and Spanish. *Quarterly Journal of Experimental Psychology*, *70*(10), 2105–2129. 10.1080/17470218.2016.1223705 27609455

[CR31] Larnyo, E., Dai, B., Nutakor, J. A., Ampon-Wireko, S., Larnyo, A., & Appiah, R. (2022). Examining the impact of socioeconomic status, demographic characteristics, lifestyle and other risk factors on adults’ cognitive functioning in developing countries: An analysis of five selected WHO SAGE Wave 1 Countries. *International Journal for Equity in Health*, *21*(1). 10.1186/s12939-022-01622-7. Article 31. PMC8876754 35216605

[CR32] Le Dorze, G., & Durocher, J. (1992). The effects of age, educational level, and stimulus length on naming in normal subjects. *Journal of Speech-Language Pathology and Audiology*, *16*(1), 21–29.

[CR33] Lefly, D. L., & Pennington, B. F. (2000). Reliability and validity of the Adult Reading History Questionnaire. *Journal of Learning Disabilities*, *33*(3), 286–296. 10.1177/002221940003300306 15505966

[CR34] Locher, F., & Pfost, M. (2019). The relation between time spent reading and reading comprehension throughout the life course. *Journal of Research in Reading*, *43*(1), 57–77. 10.1111/1467-9817.12289

[CR35] Lövdén, M., Fratiglioni, L., Glymour, M. M., Lindenberger, U., & Tucker-Drob, E. M. (2020). Education and cognitive functioning across the life span. *Psychological Science in the Public Interest*, *21*(1), 6–41. 10.1177/1529100620920576 PMC7425377 32772803

[CR36] Malcorra, B. L. C., Mota, N. B., Weissheimer, J., Schilling, L. P., Wilson, M. A., & Hübner, L. C. (2022). Reading and writing habits compensate for aging effects in speech connectedness. *NPJ Science of Learning*, *7*(1). 10.1038/s41539-022-00129-8 PMC9178018 35676305

[CR37] Martínez-Tomás, A. J., Pérez-Sánchez, M., & García-Sevilla, J. (2025a). Cognitive reserve and executive functions in healthy aging: The role of reading habits and formal education. *Applied Neuropsychology: Adult*. 10.1080/23279095.2024. [Nota: Falta o número de artigo no final do DOI se existir].

[CR38] Martínez-Tomás, C., Baciero, A., Lázaro, M., & Hinojosa, J. A. (2025b). What do pseudowords tell us about word processing? An overview. *Frontiers in Language Sciences*, *4*, 1504770.

[CR39] Mellard, D. F., Fall, E., & Woods, K. L. (2010). A path analysis of reading comprehension for adults with low literacy. *Journal of Learning Disabilities*, *43*(2), 154–165. 10.1177/0022219409359345 PMC2860173 20179309

[CR40] Mellard, D. F., Woods, K. L., Desa, M., Z. D., & Vuyk, M. A. (2015). Underlying reading-related skills and abilities among adult learners. *Journal of Learning Disabilities*, *48*(3), 310–322. 10.1177/0022219413500813 23963050

[CR41] Mora, F., Quito, R., & Macías, L. (2021). Reading comprehension and reading speed of university English language learners in Ecuador. *Journal of English Language Teaching and Applied Linguistics*, *3*(11), 11–31. 10.32996/jeltal.2021.3.11.3

[CR42] Newman, R. S., & German, D. J. (2005). Life span effects of lexical factors on oral naming. *Language and Speech*, *48*(2), 123–156. 10.1177/00238309050480020101 16411502

[CR43] Parente, M. A. M. P., Capuano, A., & Nespoulous, J. L. (1997). Ativação de significados ambíguos por jovens e idosos. *Psicologia: Reflexão e Crítica*, *10*(2), 291–308.

[CR44] Pillai, J. A., Hall, C. B., Derby, C. A., Ehrlich, A. R., Verghese, J., & Lipton, R. B. (2012). Association of crossword puzzle participation with memory decline in persons who develop dementia. *Journal of the International Neuropsychological Society*, *17*(6), 1006–1013. 10.1017/S1355617711001111 PMC3885259 22040899

[CR45] Protopapas, A. (2007). Check Vocal: A Program to Facilitate Checking the Accuracy and Response Time of Vocal Responses from DMDX. *Behavior Research Methods*, *39*, 859–862. 10.3758/BF03192979 18183901

[CR46] Provost, J. S., Brambati, S. M., Chapleau, M., & Wilson, M. A. (2016). The effect of aging on the brain network for exception word reading. *Cortex; A Journal Devoted To The Study Of The Nervous System And Behavior*, *84*, 90–100. 10.1016/j.cortex.2016.09.005 27721080

[CR47] Rakesh, G. (2024). Brain maintenance and cognitive reserve: A review of modifiable lifestyle factors. *Frontiers in Aging Neuroscience*. *[Nota: Expanda o et al. listando os autores]*.

[CR48] Rakesh, D., Lee, P. A., Gaikwad, A., & McLaughlin, K. A. (2024). Associations of socioeconomic status with cognitive function, language ability, and academic achievement in youth: A systematic review of mechanisms and protective factors. *Journal of Child Psychology and Psychiatry*. 10.1111/jcpp.14082 PMC11920614 39625804

[CR49] Rasinger, S. M. (2010). Quantitative methods: Concepts, frameworks and issues. In *Research methods in linguistics* (pp. 49–67). *[Nota: Falta os editores e a editora do livro]*.

[CR50] Riffo, B., Rojas, C., Helo, A., Véliz, M., Urzúa, P., Gutierrez, G., & Guerra, E. (2025). Reading comprehension in older adults—Effects of age, educational level, and reading habits. *Journal of Intelligence*, *13*(1). 10.3390/jintelligence13010004 PMC11765946 39852413

[CR51] Salas, N., & Pascual, M. (2023). Impact of school SES on literacy development. *Plos One*, *18*(12). 10.1371/journal.pone.0295606. Article e0295606. PMC10734911 38127961

[CR52] Schmider, E., Ziegler, M., Danay, E., Beyer, L., & Buhner, M. (2010). Is it really robust? Reinvestigating the robustness of ANOVA against violations of the normal distribution assumption. *Methodology*, *6*(4), 147–151. 10.1027/1614-2241/a000016

[CR53] Schneider, F., Marcotte, K., Brisebois, A., Townsend, S. A. M., Smidarle, A. D., Soder, R. B., Marrone, L. C. P., & Hübner, L. C. (2022). Macrostructural aspects in oral narratives in Brazilian Portuguese by left and right hemisphere stroke patients with low education and low socioeconomic status. *American Journal of Speech-Language Pathology*, *31*(3), 1319–1337. 10.1044/2021_AJSLP-21-00205 35302896

[CR54] Sörman, D. E., Ljungberg, J. K., & Rönnlund, M. (2018). Reading habits among older adults in relation to level and 15-year changes in verbal fluency and episodic recall. *Frontiers in Psychology*, *9*, 1872. 10.3389/fpsyg.2018.01872 PMC6171467 30319520

[CR55] Spieler, D. H., & Balota, D. A. (2000). Factors influencing word naming in younger and older adults. *Psychology and Aging*, *15*(2), 225–235. 10.1037/0882-7974.15.2.225 10879577

[CR56] Steffener, J. (Ed.). (2020a). Cognitive reserve: Measurement and analysis. In Y. Stern (Ed.), *Cognitive reserve*. Taylor & Francis. *[Nota: Faltam as páginas do capítulo (pp. xx-xx)]*.

[CR57] Steffener, J. (2020b). Education and age-related differences in cortical thickness and volume across the lifespan. *Neurobiology of Aging*. 10.1016/j.neurobiolaging.2020.10.034 PMC8126642 33765423

[CR58] Stern, Y. (2009). Cognitive reserve. *Neuropsychologia*, *47*(10), 2015–2028. 10.1016/j.neuropsychologia.2009.03.004 PMC2739591 19467352

[CR59] Stern, Y., Barnes, C. A., Grady, C., Jones, R. N., & Raz, N. (2019). Brain reserve, cognitive reserve, compensation, and maintenance: Operationalization, validity, and mechanisms of cognitive resilience. *Neurobiology of Aging*, *83*, 124–129. 10.1016/j.neurobiolaging.2019.03.022 PMC6859943 31732015

[CR60] Takeuchi, H., Taki, Y., Hashizume, H., Asano, K., Asano, M., Sassa, Y., Yokota, S., Kotozaki, Y., Nouchi, R., & Kawashima, R. (2016). Impact of reading habit on white matter structure: Cross-sectional and longitudinal analyses. *Neuroimage*, *133*, 378–389. 10.1016/j.neuroimage.2016.03.037 27033689

[CR61] Tan, C. Y. (2024a). Socioeconomic status and student learning: Insights from an umbrella review. *Educational Psychology Review*, *36*., Article 100. 10.1007/s10648-024-09929-3

[CR62] Tan, X. (2024b). *Advanced statistical methods in educational research: A guide for researchers*. Springer.

[CR63] The jamovi project (2023). *jamovi* (Version 2.3) [Computer software]. https://www.jamovi.org

[CR64] Walhovd, K. B., Lövdén, M., & Fjell, A. M. (2023). Timing of lifespan influences on brain and cognition. *Trends in Cognitive Sciences*, *27*(10). 10.1016/j.tics.2023.07.001 37563042

[CR65] Wang, J. (2023). The protective effect of lifetime reading on cognitive decline in late life. *International Psychogeriatrics*. *[Nota: Expanda o et al. listando os autores]*.

[CR66] Wang, X., Bakulski, K. M., Paulson, H. L., Albin, R. L., & Park, S. K. (2023). Associations of healthy lifestyle and socioeconomic status with cognitive function in US older adults. *Scientific Reports*, *13*(1). 10.1101/2023.01.24.23284961 PMC10170128 37160962

[CR67] Welcome, S. E., & Meza, R. A. (2019). Dimensions of the Adult Reading History Questionnaire and their relationships with reading ability. *Reading and Writing*, *32*(5), 1295–1317. 10.1007/s11145-018-9912-z

[CR68] Wilson, M. A., Joubert, S., Ferré, P., Belleville, S., Ansaldo, A. I., Joanette, Y., & Brambati, S. M. (2012). The role of the left anterior temporal lobe in exception word reading: Reconciling patient and neuroimaging findings. *Neuroimage*, *60*(4), 2000–2007. 10.1016/j.neuroimage.2012.02.009 22361167

[CR69] Wulff, D. U., De Deyne, S., Jones, M. N., Mata, R., & The Aging Lexicon Consortium. (2019). New perspectives on the aging lexicon. *Trends in Cognitive Sciences*, *23*(8), 686–698. 10.1016/j.tics.2019.05.003 31288976

